# Post-Treatment NLR and SII Associations with Response and Progression-Free Survival After Neoadjuvant Disitamab Vedotin Plus PD-1 Blockade in Muscle-Invasive Bladder Cancer: A Real-World Cohort Study

**DOI:** 10.3390/diagnostics16172802

**Published:** 2026-08-31

**Authors:** Yongsheng Huang, Yongpeng Ji, Xiao Yang, Qiang Zhao, Yudong Cao, Jinchao Ma, Ruijian You, Yongjie Li, Yong Yang, Peng Du, Shuo Wang

**Affiliations:** Key Laboratory of Carcinogenesis and Translational Research (Ministry of Education), Department of Urology, Peking University Cancer Hospital & Institute, Beijing 100142, China

**Keywords:** muscle-invasive bladder cancer, disitamab vedotin, immune checkpoint inhibition, neutrophil-to-lymphocyte ratio, systemic immune-inflammation index, real-world evidence

## Abstract

**Background/Objectives**: This study evaluated neoadjuvant disitamab vedotin plus programmed cell death protein 1 (PD-1) blockade and associations of post-treatment neutrophil-to-lymphocyte ratio (NLR) and systemic immune-inflammation index (SII) with response and progression-free survival (PFS) in muscle-invasive bladder cancer. **Methods**: This single-center retrospective cohort included 71 patients with cT2–T3N0M0, human epidermal growth factor receptor 2 (HER2)-expressing disease treated between September 2021 and October 2025. Logistic regression and receiver operating characteristic analyses evaluated response endpoints; Kaplan–Meier and Cox methods evaluated PFS. Benjamini–Hochberg correction was applied separately to response analyses. **Results**: Complete response (CR), objective response, and disease control rates were 66.2%, 84.5%, and 88.7%. Of 47 CRs, 5 were cystectomy-confirmed pathological CR (pCR) and 42 were clinical CR (cCR). Response associations did not remain significant after correction (minimum *q* = 0.082). AUCs were 0.659–0.708. In a two-variable Cox model based on 14 PFS events, treatment cycles (HR 0.615, 95% CI 0.419–0.903; *p* = 0.013) and SII per 100 units (HR 1.205, 95% CI 1.073–1.355; *p* = 0.002) were associated with PFS, but these estimates require cautious interpretation. Two deaths occurred. **Conclusions**: Neoadjuvant disitamab vedotin plus PD-1 blockade showed promising antitumor activity. The pooled CR endpoint may overestimate pathological response and is not directly comparable with cystectomy-confirmed pCR. Without standardized pretreatment NLR and SII measurements, reverse causality cannot be excluded. Response associations and PFS findings based on 14 events require cautious interpretation and prospective validation.

## 1. Introduction

Bladder cancer is a major global health burden, and muscle-invasive bladder cancer (MIBC) carries a substantial risk of metastatic recurrence and death [1,2]. Cisplatin-based neoadjuvant chemotherapy followed by radical cystectomy remains a standard approach for eligible patients. However, renal dysfunction, comorbidity, treatment intolerance, and heterogeneous tumor response restrict its use, creating a need for effective, better-tolerated perioperative strategies [3,4,5].

Disitamab vedotin is a human epidermal growth factor receptor 2 (HER2)-directed antibody–drug conjugate that delivers monomethyl auristatin E to HER2-expressing tumor cells [6,7]. Clinical and real-world studies have reported encouraging activity of disitamab vedotin alone or with programmed cell death protein 1 (PD-1) blockade in urothelial carcinoma, including the neoadjuvant setting [8,9,10,11,12,13,14,15]. Nevertheless, accessible markers that characterize response after treatment remain limited.

Peripheral blood inflammatory and nutritional indices are inexpensive and routinely available. The neutrophil-to-lymphocyte ratio (NLR), systemic immune-inflammation index (SII), and prognostic nutritional index (PNI) have been associated with treatment response or prognosis in bladder and urothelial cancers [16,17,18,19,20]. Because values measured after neoadjuvant therapy may be downstream correlates of response, toxicity, treatment duration, or intercurrent inflammation, they should be considered post-treatment association measures rather than pretreatment predictive biomarkers.

Therefore, this study evaluated the activity of neoadjuvant disitamab vedotin plus PD-1 blockade in a single-center, real-world cohort of patients with cT2–T3N0M0, HER2-expressing MIBC. This study also explored associations of clinicopathological characteristics and post-treatment inflammatory indices with complete response (CR), objective response rate (ORR), and progression-free survival (PFS). Disease control rate (DCR) and overall survival (OS) were summarized descriptively because of sparse non-DCR outcomes and deaths.

## 2. Materials and Methods

### 2.1. Study Design and Population

**Figure 1:**
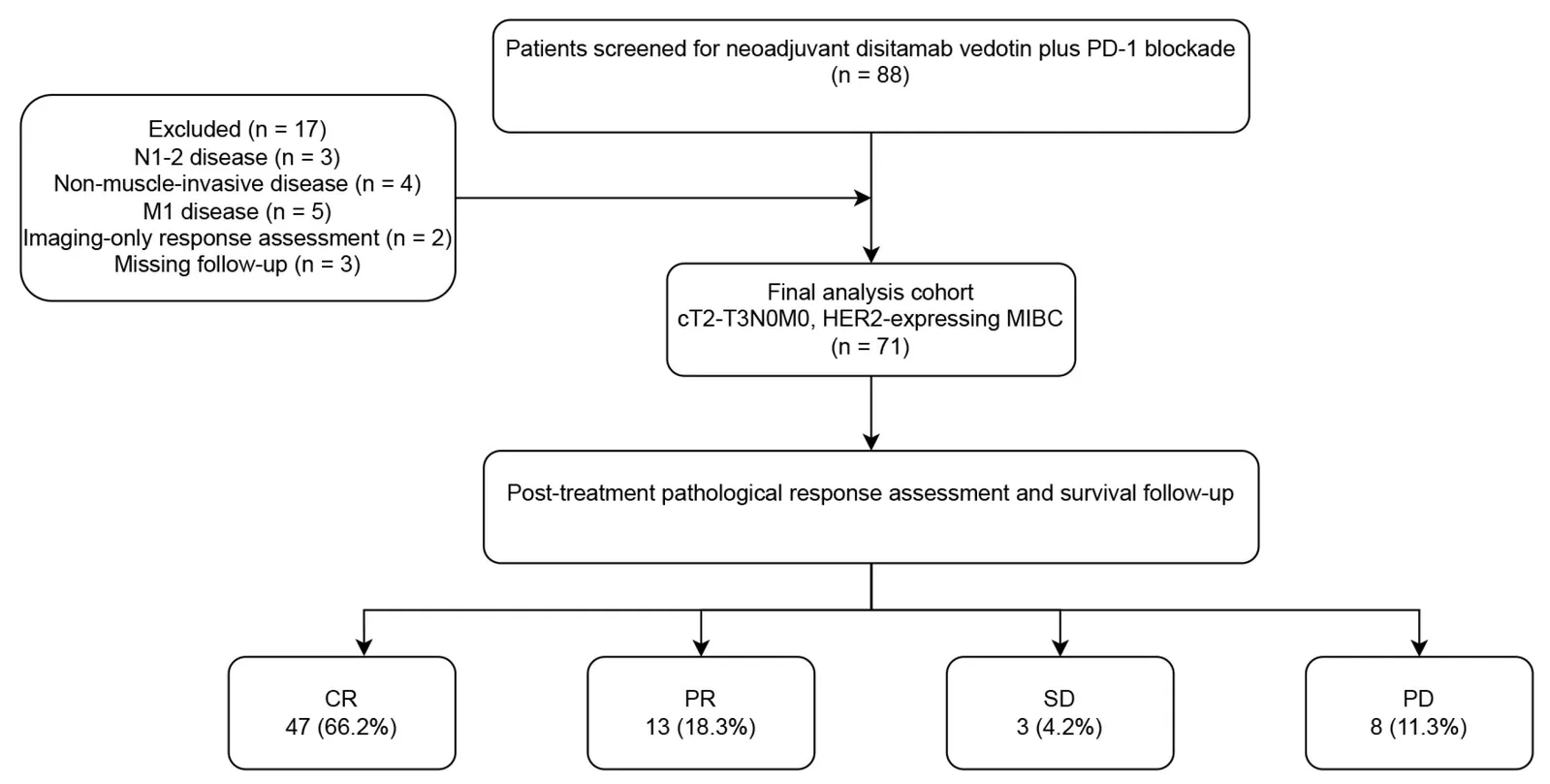
Study cohort selection and response classification. CR, complete response; PR, partial response; SD, stable disease; PD, progressive disease; HER2, human epidermal growth factor receptor 2; MIBC, muscle-invasive bladder cancer; PD-1, programmed cell death protein 1.

This retrospective cohort study included patients treated in the Department of Urology at Peking University Cancer Hospital between September 2021 and October 2025. Follow-up data were updated through 17 May 2026. The study flow is summarized in Figure 1.

Eligible patients had pathologically confirmed MIBC staged cT2–T3N0M0 before neoadjuvant therapy; HER2 expression of IHC 1+, 2+, or 3+ in pretreatment tumor tissue; no previous neoadjuvant systemic chemotherapy, targeted therapy, or immune checkpoint inhibition; maximal transurethral resection of bladder tumor before treatment; neoadjuvant disitamab vedotin combined with PD-1 blockade; and an available post-treatment pathological assessment. Treatment cycles were individualized according to response, toxicity, patient preference, surgical scheduling, bladder-preservation goals, and multidisciplinary assessment. Patients with nodal or distant metastases, non–muscle-invasive disease, another active malignancy, incomplete essential data, or an acute infection or inflammatory condition affecting the blood count were excluded.

### 2.2. Data Collection and Laboratory Indices

Demographic, clinical, pathological, treatment, and outcome data were obtained from electronic medical records. Recorded variables included age, sex, comorbidities, smoking and alcohol history, body mass index, body surface area, disease presentation (newly diagnosed vs. recurrent), unifocal or multifocal disease, clinical T stage, treatment cycles, concurrent radiotherapy, HER2 IHC intensity, combined positive score (CPS), Ki-67 index, and post-treatment hematologic and biochemical measurements. Individual PD-1 inhibitors were not compared because treatment assignment was nonrandomized.

Laboratory values were collected after completion of the analyzed neoadjuvant treatment period and before response assessment. NLR was calculated as absolute neutrophil count divided by absolute lymphocyte count. SII was calculated as platelet count multiplied by absolute neutrophil count and divided by absolute lymphocyte count. PNI was calculated as serum albumin plus five times the absolute lymphocyte count. Because the indices were measured after treatment, temporal precedence over response could not be assumed.

### 2.3. Response and Survival Endpoints

The primary endpoint was a pragmatic real-world composite CR endpoint after neoadjuvant therapy, comprising pathological complete response (pCR) and clinical complete response (cCR) according to the assessment pathway. For patients undergoing radical cystectomy, pCR was defined as no viable residual tumor in the cystectomy specimen (ypT0N0). For patients who did not undergo radical cystectomy, cCR required negative pathological findings on repeat transurethral resection of bladder tumor, negative urine cytology, and no residual or metastatic disease on imaging. The pCR and cCR components were pooled for analysis but were not considered clinically or biologically equivalent.

Patients not meeting CR criteria were classified as having partial response (PR), stable disease (SD), or progressive disease (PD) based on integrated pathological, cystoscopic, and radiographic assessment. ORR was defined as CR plus PR; DCR was defined as CR plus PR plus SD.

Progression-free survival (PFS) was measured from the first dose of neoadjuvant therapy to local progression, recurrence, distant metastasis, or death from any cause. Patients without an event were censored at their last assessment. Overall survival (OS) was measured from the first dose to death from any cause. Because only two deaths occurred, OS was summarized descriptively and no inferential OS model or group comparison was performed.

### 2.4. Statistical Analysis

Statistical analyses and figure generation were performed using IBM SPSS Statistics 27 and R version 4.4.1. Continuous variables are presented as mean ± standard deviation (SD) and were compared using the Mann–Whitney U test. Categorical variables are presented as numbers and percentages and were compared using Fisher’s exact test. Analyses were based on available cases without imputation. Univariable logistic regression was used to analyze factors associated with CR and ORR. Because of the limited number of outcome events, no multivariable response model was constructed, and DCR was summarized descriptively. ROC curves were used to evaluate the discriminatory ability of relevant indicators for CR and ORR. AUC 95% confidence intervals were calculated using DeLong’s method. Benjamini–Hochberg correction was applied separately to multiple comparisons in the univariable logistic regression analyses for CR and ORR, with both *p* values and *q* values reported. Univariable and multivariable Cox regression analyses were used to evaluate factors associated with PFS, and Kaplan–Meier methods were used to estimate PFS and OS. Treatment cycles and SII were included in the multivariable Cox model. The proportional-hazards assumption was assessed using Schoenfeld residuals. SII was scaled per 100-unit increase in both logistic and Cox regression analyses. Because NLR and SII may be collinear, they were not simultaneously included in the same multivariable model. Because few deaths occurred, OS was analyzed descriptively. All statistical tests were two-sided, with *p* < 0.05 considered statistically significant; for analyses with Benjamini–Hochberg correction, *q* < 0.05 was considered statistically significant after adjustment. All inferential analyses were interpreted as exploratory and hypothesis-generating.

## 3. Results

### 3.1. Cohort and Treatment Response

**Figure 2:**
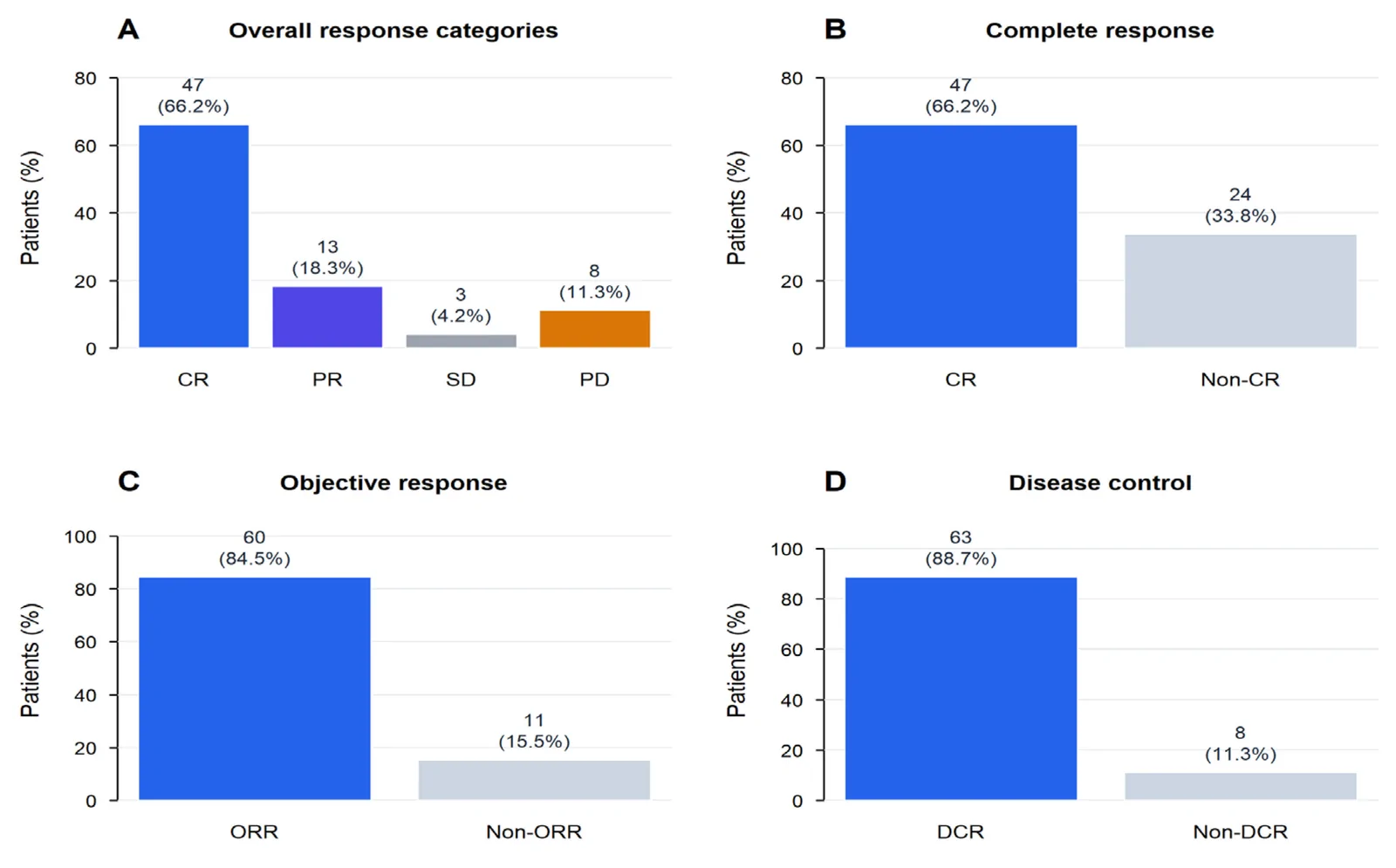
Distribution of treatment responses after neoadjuvant therapy. (**A**) Complete, partial, stable, and progressive disease categories. (**B**) Complete response versus non-complete response. (**C**) Objective response versus nonresponse. (**D**) Disease control versus no disease control. CR, complete response; PR, partial response; SD, stable disease; PD, progressive disease; ORR, objective response rate; DCR, disease control rate.

Among 88 screened patients, 17 were excluded: three had N1–2 disease, four had non–muscle-invasive disease, five had M1 disease, two had imaging-only response assessment without pathological confirmation, and three lacked follow-up data. The final cohort comprised 71 patients. Forty-seven patients achieved CR (66.2%), 13 had PR (18.3%), three had SD (4.2%), and eight had PD (11.3%). ORR was 84.5% (60/71), and DCR was 88.7% (63/71) (Figure 2). Among the 47 patients who achieved CR, 5 had cystectomy-confirmed pCR and 42 met the cCR criteria.

The mean age was 62.92 ± 10.03 years; 55 patients (77.5%) were male. Baseline demographic and clinical characteristics were generally similar between the CR and non-CR groups (Table 1). The CR group received more treatment cycles (5.47 ± 1.23 vs. 4.75 ± 1.80; *p* = 0.047) and had higher CPS (16.16 ± 23.73 vs. 8.00 ± 15.17; *p* = 0.014). Post-treatment creatinine, NLR, and SII were lower in the CR group. These comparisons were unadjusted.

Supplementary Table S1 summarizes characteristics by ORR status. Compared with nonresponders, patients achieving ORR received more treatment cycles (5.43 ± 1.31 vs. 4.09 ± 1.87; *p* = 0.018) and had lower post-treatment NLR (2.64 ± 1.05 vs. 3.64 ± 1.61; *p* = 0.030). These comparisons were unadjusted. Supplementary Table S2 summarizes characteristics by DCR status. Patients achieving DCR had lower post-treatment NLR than those who did not (2.69 ± 1.20 vs. 3.58 ± 0.79); however, because only eight patients did not achieve disease control, this finding was considered descriptive.

### 3.2. HER2 Expression and Response

CR rates were 75.0% (6/8), 63.0% (29/46), and 70.6% (12/17) in the HER2 IHC 1+, 2+, and 3+ groups, respectively (*p* = 0.803). Corresponding ORRs were 75.0%, 82.6%, and 94.1% (*p* = 0.320), and DCRs were 87.5%, 87.0%, and 94.1%, respectively; DCR was summarized descriptively (Table 2). No statistically significant CR or ORR differences were detected, but the small IHC 1+ and 3+ subgroups precluded interpreting these findings as equivalent efficacy across HER2 levels.

### 3.3. Factors Associated with CR and ORR

**Figure 3:**
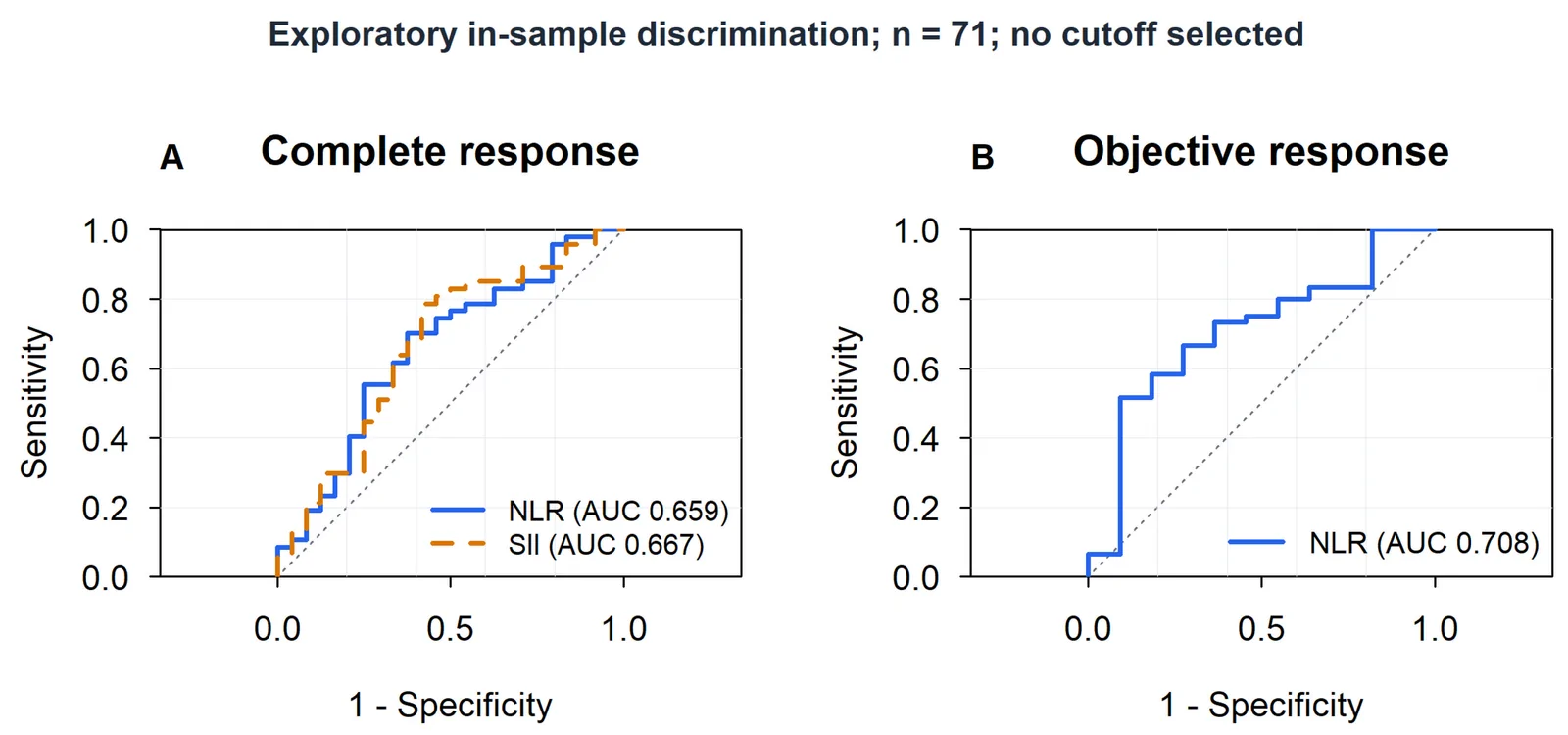
Exploratory receiver operating characteristic curves for in-sample response discrimination. (**A**) Post-treatment NLR and SII for complete response. (**B**) Post-treatment NLR for objective response. No cutoff was selected, and the curves were not internally or externally validated. NLR, neutrophil-to-lymphocyte ratio; SII, systemic immune-inflammation index; AUC, area under the curve.

Univariable logistic regression results for candidate variables are shown in Table 3. Higher post-treatment NLR (OR 0.585; 95% CI 0.368–0.930; *p* = 0.023) and SII (per 100 units: OR 0.834; 95% CI 0.710–0.980; *p* = 0.028) were associated with lower odds of CR; however, neither met the BH-adjusted *q* < 0.05 threshold (both *q* = 0.125). Their discrimination for CR was limited (NLR AUC, 0.659; 95% CI, 0.520–0.797; SII AUC, 0.667; 95% CI, 0.526–0.807) (Figure 3A).

For ORR, more treatment cycles (OR 1.848; 95% CI 1.164–2.933; *p* = 0.009), lower post-treatment NLR (OR 0.523; 95% CI 0.301–0.911; *p* = 0.022), and lower post-treatment SII per 100 units (OR 0.829; 95% CI 0.702–0.979; *p* = 0.027) met the nominal *p* < 0.05 threshold; each had BH-adjusted *q* = 0.082. No multivariable ORR model was retained because only 11 patients were nonresponders. NLR yielded an AUC of 0.708 (DeLong 95% CI 0.538–0.877) for ORR (Figure 3B). DCR regression and ROC analyses were not performed because only eight patients lacked disease control.

### 3.4. Follow-Up and Survival Outcomes

The median observed follow-up was 24.90 months (IQR, 12.01–37.21). Fourteen PFS events occurred: eight patients experienced PD during or immediately after neoadjuvant therapy, five subsequently developed recurrence, progression, or metastasis, and one died without a previously recorded progression event. Estimated PFS rates at 12, 24, and 36 months were 88.7%, 76.2%, and 76.2%, respectively. Two deaths occurred; estimated OS rates at 12, 24, and 36 months were 98.3%, 96.4%, and 96.4%, respectively. Because only two deaths occurred, OS was summarized descriptively and no reliable inferential conclusions were drawn.

In univariable Cox regression analyses, treatment cycles and SII were associated with PFS (Table 4). In the two-variable Cox model, each additional treatment cycle was associated with a lower hazard of a PFS event (HR 0.615; 95% CI 0.419–0.903; *p* = 0.013), whereas each 100-unit increase in SII was associated with a higher hazard (HR 1.205; 95% CI 1.073–1.355; *p* = 0.002). The proportional-hazards assumption was not violated (global *p* = 0.198). These associations should not be interpreted causally because treatment exposure was individualized and SII was measured after treatment.

## 4. Discussion

In this single-center real-world cohort, neoadjuvant disitamab vedotin plus PD-1 blockade was associated with a CR rate of 66.2%, ORR of 84.5%, and DCR of 88.7% in patients with cT2–T3N0M0, HER2-expressing MIBC. Post-treatment NLR and SII showed nominal associations with response, but neither met the BH-adjusted *q* < 0.05 threshold. In a two-variable Cox model based on only 14 PFS events, treatment cycles and post-treatment SII were associated with PFS; these estimates are hypothesis-generating and require prospective external validation.

The observed activity is consistent with emerging reports of disitamab vedotin combined with immune checkpoint inhibition [8,9,10,11,12,13,14,15,21,22], although cross-study comparisons are constrained by different eligibility criteria, response definitions, surgical rates, schedules, and follow-up. No response difference was detected across HER2 IHC levels, but the IHC 1+ and 3+ subgroups were very small; nonsignificance therefore does not establish equivalent efficacy [23,24].

The treatment-cycle associations cannot show that additional cycles improve response or PFS. Treatment duration was individualized, and patients with early progression cannot complete more cycles, producing confounding by indication and guarantee-time bias [25,26].

NLR and SII are inexpensive and routinely available, but in this study they were measured after treatment and before response assessment [27,28,29,30,31,32]. They may reflect response, toxicity, treatment duration, or intercurrent inflammation, creating reverse causality; inconsistent baseline measurements precluded paired pre- and post-treatment analysis. Moreover, none of the nominal response associations met *q* < 0.05 after BH correction, and AUCs of 0.659–0.708 indicated only modest, unvalidated in-sample discrimination. These results do not support pretreatment prediction, cutoffs, or stand-alone clinical decisions.

The CR endpoint was a pragmatic composite of cystectomy-confirmed pCR and cCR based on negative repeat local assessment and imaging. Only 5 of 47 CRs were cystectomy-confirmed pCR, whereas 42 were cCR. Because these components are not equivalent and cCR may miss occult residual disease because of sampling and restaging limitations, pooling them may overestimate true pathological response; therefore, the 66.2% CR rate should not be compared directly with studies using uniform cystectomy pathology [33,34].

The main limitations are the retrospective single-center design, small sample and event counts, heterogeneous response pathways, nonrandom treatment exposure, multiple analyses, unvalidated ROC estimates, and only two deaths. Because standardized baseline NLR and SII were unavailable, the study cannot distinguish whether post-treatment values are independent biological correlates or downstream consequences of response, toxicity, or treatment duration. DCR and OS were therefore descriptive, and no multivariable ORR model was retained. Prospective multicenter studies should standardize pCR and cCR assessment, collect pretreatment and serial biomarkers, prespecify models and cutoffs, and evaluate bladder-intact, PFS, OS, and patient-reported outcomes.

## 5. Conclusions

Neoadjuvant disitamab vedotin plus PD-1 blockade showed promising activity in this selected real-world cohort of HER2-expressing MIBC. Post-treatment NLR and SII showed nominal associations with response, but none met the BH-adjusted *q* < 0.05 threshold and their in-sample ROC discrimination was modest. In a parsimonious Cox model with only 14 PFS events, treatment cycles and post-treatment SII were associated with PFS; the cycles’ association is susceptible to indication and guarantee-time bias and should not be interpreted causally. These post-treatment indices are not pretreatment predictive biomarkers. Prospective multicenter validation with standardized response assessment and serial biomarker measurement is required before clinical implementation.

## Figures and Tables

**Table 1:** Patient characteristics and post-treatment laboratory indices stratified by complete response status.

Variable	Total (*n* = 71)	CR (*n* = 47)	Non-CR (*n* = 24)	*p* Value
Age, years	62.92 ± 10.03	62.62 ± 8.81	63.50 ± 12.27	0.784
Sex				
Female	16 (22.5)	12 (25.5)	4 (16.7)	0.551
Male	55 (77.5)	35 (74.5)	20 (83.3)	
Hematuria				
No	17 (23.9)	13 (27.7)	4 (16.7)	0.386
Yes	54 (76.1)	34 (72.3)	20 (83.3)	
Hypertension				
No	43 (60.6)	27 (57.4)	16 (66.7)	0.608
Yes	28 (39.4)	20 (42.6)	8 (33.3)	
Diabetes				
No	61 (85.9)	40 (85.1)	21 (87.5)	1.000
Yes	10 (14.1)	7 (14.9)	3 (12.5)	
Coronary heart disease				
No	65 (91.5)	44 (93.6)	21 (87.5)	0.399
Yes	6 (8.5)	3 (6.4)	3 (12.5)	
Smoking history				
No	24 (33.8)	13 (27.7)	11 (45.8)	0.185
Yes	47 (66.2)	34 (72.3)	13 (54.2)	
Alcohol history				
No	51 (71.8)	32 (68.1)	19 (79.2)	0.409
Yes	20 (28.2)	15 (31.9)	5 (20.8)	
Recurrent disease				
No	53 (74.6)	35 (74.5)	18 (75.0)	1.000
Yes	18 (25.4)	12 (25.5)	6 (25.0)	
Multiple tumors				
No	20 (28.2)	11 (23.4)	9 (37.5)	0.268
Yes	51 (71.8)	36 (76.6)	15 (62.5)	
Body mass index	24.80 ± 3.77	24.63 ± 3.98	25.14 ± 3.37	0.473
Body surface area, m^2^	1.80 ± 0.18	1.80 ± 0.21	1.81 ± 0.13	0.922
Clinical T stage				
T2	58 (81.7)	41 (87.2)	17 (70.8)	0.112
T3	13 (18.3)	6 (12.8)	7 (29.2)	
Neoadjuvant cycles	5.23 ± 1.48	5.47 ± 1.23	4.75 ± 1.80	0.047
Concurrent radiotherapy				
No	69 (97.2)	45 (95.7)	24 (100.0)	0.546
Yes	2 (2.8)	2 (4.3)	0 (0.0)	
CPS	13.49 ± 21.51	16.16 ± 23.73	8.00 ± 15.17	0.014
Ki-67, %	50.30 ± 25.33	54.74 ± 24.29	39.75 ± 26.17	0.200
White blood cells	6.34 ± 1.84	6.11 ± 1.81	6.80 ± 1.84	0.148
Lymphocytes	1.57 ± 0.53	1.61 ± 0.54	1.51 ± 0.51	0.477
Neutrophils	4.09 ± 1.48	3.86 ± 1.43	4.53 ± 1.51	0.071
Platelets	237.92 ± 70.97	233.55 ± 72.42	246.46 ± 68.74	0.346
Albumin	43.07 ± 3.39	43.51 ± 2.62	42.19 ± 4.48	0.584
Monocytes	0.40 ± 0.14	0.38 ± 0.12	0.44 ± 0.17	0.107
Hemoglobin	133.56 ± 19.11	134.55 ± 15.47	131.63 ± 25.04	0.932
Creatinine	81.44 ± 31.99	76.21 ± 22.05	91.67 ± 44.47	0.036
Uric acid	346.06 ± 91.76	350.70 ± 78.98	336.96 ± 114.09	0.455
Glucose	6.29 ± 2.26	6.36 ± 2.14	6.14 ± 2.52	0.697
Lactate dehydrogenase	204.79 ± 40.74	211.66 ± 41.65	191.33 ± 36.00	0.138
Total cholesterol	4.86 ± 1.00	4.90 ± 1.06	4.76 ± 0.89	0.610
NLR	2.79 ± 1.19	2.55 ± 0.99	3.27 ± 1.41	0.030
SII	667.51 ± 371.56	590.07 ± 273.07	819.16 ± 484.55	0.023
PNI	50.94 ± 4.69	51.56 ± 3.88	49.71 ± 5.87	0.365

Data are mean ± standard deviation or *n* (%). CR, complete response; CPS, combined positive score; NLR, neutrophil-to-lymphocyte ratio; SII, systemic immune-inflammation index; PNI, prognostic nutritional index.

**Table 2:** Association between HER2 immunohistochemical expression and treatment response.

Endpoint/HER2 IHC	Overall	Response	Nonresponse	*p* Value
Complete response				
1+	8 (11.3)	6 (12.8)	2 (8.3)	0.803
2+	46 (64.8)	29 (61.7)	17 (70.8)	
3+	17 (23.9)	12 (25.5)	5 (20.8)	
Objective response				
1+	8 (11.3)	6 (10.0)	2 (18.2)	0.320
2+	46 (64.8)	38 (63.3)	8 (72.7)	
3+	17 (23.9)	16 (26.7)	1 (9.1)	
Disease control				
1+	8 (11.3)	7 (11.1)	1 (12.5)	-
2+	46 (64.8)	40 (63.5)	6 (75.0)	
3+	17 (23.9)	16 (25.4)	1 (12.5)	

Data are *n* (%). HER2, human epidermal growth factor receptor 2; IHC, immunohistochemistry. DCR was summarized descriptively; no hypothesis test was performed.

**Table 3:** Exploratory univariable logistic regression analyses of complete and objective response.

Endpoint/Variable	Univariable OR (95% CI)	Nominal *p* Value	BH *q* Value
Complete response			
Treatment cycles	1.411 (0.988–2.014)	0.058	0.174
CPS	1.023 (0.988–1.060)	0.204	0.306
NLR	0.585 (0.368–0.930)	0.023	0.125
SII, per 100 units	0.834 (0.710–0.980)	0.028	0.125
PNI	1.090 (0.977–1.216)	0.125	0.225
T3 vs. T2	0.355 (0.104–1.214)	0.099	0.222
HER2 2+ vs. 1+	0.569 (0.103–3.140)	0.517	0.665
HER2 3+ vs. 1+	0.800 (0.118–5.404)	0.819	0.819
Age, per year	0.991 (0.942–1.042)	0.724	0.815
Objective response			
Treatment cycles	1.848 (1.164–2.933)	0.009	0.082
CPS	1.001 (0.969–1.034)	0.949	0.949
NLR	0.523 (0.301–0.911)	0.022	0.082
SII, per 100 units	0.829 (0.702–0.979)	0.027	0.082
PNI	1.082 (0.949–1.234)	0.237	0.355
T3 vs. T2	0.309 (0.075–1.275)	0.104	0.235
HER2 2+ vs. 1+	1.583 (0.269–9.320)	0.611	0.688
HER2 3+ vs. 1+	5.333 (0.405–70.194)	0.203	0.355
Age, per year	1.023 (0.964–1.087)	0.451	0.579

OR, odds ratio; CI, confidence interval; CPS, combined positive score; NLR, neutrophil-to-lymphocyte ratio; SII, systemic immune-inflammation index; PNI, prognostic nutritional index. BH *q* values were calculated separately for the CR and ORR analyses; no *q* value was below 0.05.

**Table 4:** Cox regression analyses of progression-free survival.

Variable	Univariable HR (95% CI)	*p* Value	Multivariable HR (95% CI)	*p* Value
Treatment cycles	0.641 (0.446–0.923)	0.017	0.615 (0.419–0.903)	0.013
SII, per 100 units	1.206 (1.072–1.356)	0.002	1.205 (1.073–1.355)	0.002

HR, hazard ratio; CI, confidence interval; SII, systemic immune-inflammation index.

## Data Availability

The data supporting the findings of this study are available from the corresponding author upon reasonable request. The data are not publicly available due to patient privacy and ethical restrictions.

## Supplementary Materials

The following supporting information can be downloaded at: https://www.mdpi.com/article/10.3390/diagnostics16172802/s1, Table S1: Patient characteristics and post-treatment laboratory indices by objective response status; Table S2: Patient characteristics and post-treatment laboratory indices by disease control status.
