# Supplementary material for: Post-Treatment NLR and SII Associations with Response and Progression-Free Survival After Neoadjuvant Disitamab Vedotin Plus PD-1 Blockade in Muscle-Invasive Bladder Cancer: A Real-World Cohort Study
Source: Diagnostics (Basel). 2026 Aug 31;16(17):2802. doi: 10.3390/diagnostics16172802 (PMC13565595; doi:10.3390/diagnostics16172802)
Supplement: Supplementary file 1 [file diagnostics-16-02802-s001.zip › Supplementary Table S1(ORR).pdf]

**Supplementary Table S1. Patient characteristics and post-treatment laboratory indices by objective response status**

| Variable | Total (n = 71) | ORR (n = 60) | Non-ORR (n = 11) | Nominal P value |
|---|---|---|---|---|
| Age, years | 62.92 ± 10.03 | 63.30 ± 10.33 | 60.82 ± 8.29 | 0.200 |
| **Sex** | | | | |
| Female | 16 (22.5) | 15 (25.0) | 1 (9.1) | 0.436 |
| Male | 55 (77.5) | 45 (75.0) | 10 (90.9) | |
| **Hematuria** | | | | |
| No | 17 (23.9) | 14 (23.3) | 3 (27.3) | 0.718 |
| Yes | 54 (76.1) | 46 (76.7) | 8 (72.7) | |
| **Hypertension** | | | | |
| No | 43 (60.6) | 36 (60.0) | 7 (63.6) | 1.000 |
| Yes | 28 (39.4) | 24 (40.0) | 4 (36.4) | |
| **Diabetes** | | | | |
| No | 61 (85.9) | 51 (85.0) | 10 (90.9) | 1.000 |
| Yes | 10 (14.1) | 9 (15.0) | 1 (9.1) | |
| **Coronary heart disease** | | | | |
| No | 65 (91.5) | 55 (91.7) | 10 (90.9) | 1.000 |
| Yes | 6 (8.5) | 5 (8.3) | 1 (9.1) | |
| **Smoking history** | | | | |
| No | 24 (33.8) | 20 (33.3) | 4 (36.4) | 1.000 |
| Yes | 47 (66.2) | 40 (66.7) | 7 (63.6) | |
| **Alcohol history** | | | | |
| No | 51 (71.8) | 43 (71.7) | 8 (72.7) | 1.000 |
| Yes | 20 (28.2) | 17 (28.3) | 3 (27.3) | |
| **Recurrent disease** | | | | |
| No | 53 (74.6) | 45 (75.0) | 8 (72.7) | 1.000 |
| Yes | 18 (25.4) | 15 (25.0) | 3 (27.3) | |
| **Multiple tumors** | | | | |
| No | 20 (28.2) | 16 (26.7) | 4 (36.4) | 0.491 |
| Yes | 51 (71.8) | 44 (73.3) | 7 (63.6) | |
| Body mass index | 24.80 ± 3.77 | 24.55 ± 3.81 | 26.16 ± 3.43 | 0.121 |
| Body surface area, $m^2$ | 1.80 ± 0.18 | 1.79 ± 0.20 | 1.84 ± 0.10 | 0.469 |
| **Clinical T stage** | | | | |
| T2 | 58 (81.7) | 51 (85.0) | 7 (63.6) | 0.108 |
| T3 | 13 (18.3) | 9 (15.0) | 4 (36.4) | |
| Neoadjuvant cycles | 5.23 ± 1.48 | 5.43 ± 1.31 | 4.09 ± 1.87 | 0.018 |

| Variable | Total (n = 71) | ORR (n = 60) | Non-ORR (n = 11) | Nominal P value |
|---|---|---|---|---|
| **Concurrent radiotherapy** | | | | |
| No | 69 (97.2) | 58 (96.7) | 11 (100.0) | 1.000 |
| Yes | 2 (2.8) | 2 (3.3) | 0 (0.0) | |
| CPS | 13.49 ± 21.51 | 13.58 ± 22.19 | 13.10 ± 19.19 | 0.429 |
| Ki-67, % | 50.30 ± 25.33 | 51.22 ± 25.81 | 45.00 ± 25.17 | 0.731 |
| White blood cells | 6.34 ± 1.84 | 6.29 ± 1.80 | 6.62 ± 2.09 | 0.662 |
| Lymphocytes | 1.57 ± 0.53 | 1.62 ± 0.53 | 1.34 ± 0.47 | 0.137 |
| Neutrophils | 4.09 ± 1.48 | 4.01 ± 1.46 | 4.53 ± 1.56 | 0.227 |
| Platelets | 237.92 ± 70.97 | 235.52 ± 68.80 | 251.00 ± 84.31 | 0.557 |
| Albumin | 43.07 ± 3.39 | 43.14 ± 3.22 | 42.68 ± 4.40 | 0.793 |
| Monocytes | 0.40 ± 0.14 | 0.40 ± 0.14 | 0.41 ± 0.19 | 0.943 |
| Hemoglobin | 133.56 ± 19.11 | 133.07 ± 17.43 | 136.27 ± 27.48 | 0.320 |
| Creatinine | 81.44 ± 31.99 | 78.25 ± 23.07 | 98.82 ± 60.26 | 0.139 |
| Uric acid | 346.06 ± 91.76 | 353.53 ± 94.50 | 305.27 ± 63.88 | 0.150 |
| Glucose | 6.29 ± 2.26 | 6.17 ± 1.95 | 6.93 ± 3.57 | 0.353 |
| Lactate dehydrogenase | 204.79 ± 40.74 | 207.25 ± 41.90 | 191.36 ± 32.05 | 0.530 |
| Total cholesterol | 4.86 ± 1.00 | 4.87 ± 1.01 | 4.77 ± 0.99 | 0.861 |
| NLR | 2.79 ± 1.19 | 2.64 ± 1.05 | 3.64 ± 1.61 | 0.030 |
| SII | 667.51 ± 371.56 | 618.81 ± 284.13 | 933.15 ± 631.07 | 0.110 |
| PNI | 50.94 ± 4.69 | 51.22 ± 4.54 | 49.38 ± 5.41 | 0.400 |

Note: Data are presented as mean ± standard deviation or n (%). Nominal P values were calculated using the Wilcoxon rank-sum test for continuous variables and Fisher's exact test for categorical variables. P values were not adjusted for multiple comparisons and should be interpreted as exploratory. Abbreviations: ORR, objective response rate; CPS, combined positive score; NLR, neutrophil-to-lymphocyte ratio; SII, systemic immune-inflammation index; PNI, prognostic nutritional index.
