# Supplementary material for: Post-Treatment NLR and SII Associations with Response and Progression-Free Survival After Neoadjuvant Disitamab Vedotin Plus PD-1 Blockade in Muscle-Invasive Bladder Cancer: A Real-World Cohort Study
Source: Diagnostics (Basel). 2026 Aug 31;16(17):2802. doi: 10.3390/diagnostics16172802 (PMC13565595; doi:10.3390/diagnostics16172802)
Supplement: Supplementary file 1 [file diagnostics-16-02802-s001.zip › Supplementary Table S2 (DCR).pdf]

Supplementary Table S2. Patient characteristics and post-treatment laboratory indices by disease control status

Variable	Total (n = 71)	DCR (n = 63)	Non-DCR (n = 8)
Age, years	62.92 ± 10.03	63.14 ± 10.21	61.12 ± 8.94
**Sex**			
Female	16 (22.5)	15 (23.8)	1 (12.5)
Male	55 (77.5)	48 (76.2)	7 (87.5)
**Hematuria**			
No	17 (23.9)	14 (22.2)	3 (37.5)
Yes	54 (76.1)	49 (77.8)	5 (62.5)
**Hypertension**			
No	43 (60.6)	39 (61.9)	4 (50.0)
Yes	28 (39.4)	24 (38.1)	4 (50.0)
**Diabetes**			
No	61 (85.9)	53 (84.1)	8 (100.0)
Yes	10 (14.1)	10 (15.9)	0 (0.0)
**Coronary heart disease**			
No	65 (91.5)	58 (92.1)	7 (87.5)
Yes	6 (8.5)	5 (7.9)	1 (12.5)
**Smoking history**			
No	24 (33.8)	21 (33.3)	3 (37.5)
Yes	47 (66.2)	42 (66.7)	5 (62.5)
**Alcohol history**			
No	51 (71.8)	44 (69.8)	7 (87.5)
Yes	20 (28.2)	19 (30.2)	1 (12.5)
**Recurrent disease**			
No	53 (74.6)	47 (74.6)	6 (75.0)
Yes	18 (25.4)	16 (25.4)	2 (25.0)
**Multiple tumors**			
No	20 (28.2)	16 (25.4)	4 (50.0)
Yes	51 (71.8)	47 (74.6)	4 (50.0)
Body mass index	24.80 ± 3.77	24.65 ± 3.77	25.97 ± 3.84
Body surface area, m^2	1.80 ± 0.18	1.80 ± 0.19	1.83 ± 0.10
**Clinical T stage**			
T2	58 (81.7)	53 (84.1)	5 (62.5)
T3	13 (18.3)	10 (15.9)	3 (37.5)
Neoadjuvant cycles	5.23 ± 1.48	5.38 ± 1.30	4.00 ± 2.20

Variable	Total (n = 71)	DCR (n = 63)	Non-DCR (n = 8)
**Concurrent radiotherapy**			
No	69 (97.2)	61 (96.8)	8 (100.0)
Yes	2 (2.8)	2 (3.2)	0 (0.0)
CPS	13.49 ± 21.51	14.09 ± 22.43	10.00 ± 15.72
Ki-67, %	50.30 ± 25.33	49.92 ± 26.03	53.33 ± 23.09
White blood cells	6.34 ± 1.84	6.31 ± 1.82	6.63 ± 2.07
Lymphocytes	1.57 ± 0.53	1.61 ± 0.53	1.29 ± 0.39
Neutrophils	4.09 ± 1.48	4.03 ± 1.50	4.54 ± 1.32
Platelets	237.92 ± 70.97	237.56 ± 68.92	240.75 ± 91.05
Albumin	43.07 ± 3.39	43.13 ± 3.18	42.60 ± 5.03
Monocytes	0.40 ± 0.14	0.40 ± 0.14	0.45 ± 0.18
Hemoglobin	133.56 ± 19.11	133.98 ± 17.58	130.25 ± 30.02
Creatinine	81.44 ± 31.99	78.19 ± 22.68	107.00 ± 69.54
Uric acid	346.06 ± 91.76	351.97 ± 92.65	299.50 ± 73.29
Glucose	6.29 ± 2.26	6.16 ± 1.93	7.30 ± 4.09
Lactate dehydrogenase	204.79 ± 40.74	207.71 ± 40.96	181.75 ± 32.50
Total cholesterol	4.86 ± 1.00	4.88 ± 1.03	4.69 ± 0.81
NLR	2.79 ± 1.19	2.69 ± 1.20	3.58 ± 0.79
SII	667.51 ± 371.56	636.53 ± 326.79	911.47 ± 598.36
PNI	50.94 ± 4.69	51.17 ± 4.45	49.07 ± 6.32

Note: Data are presented as mean ± standard deviation or n (%). DCR characteristics are presented descriptively, and no hypothesis tests were performed. Abbreviations: DCR, disease control rate; CPS, combined positive score; NLR, neutrophil-to-lymphocyte ratio; SII, systemic immune-inflammation index; PNI, prognostic nutritional index.
